# Scalable Community-Based Exercise to Support Physical Activity in Cancer Care: A Hybrid Effectiveness-Implementation Study

**DOI:** 10.3390/cancers18162641

**Published:** 2026-08-16

**Authors:** Margaret L. McNeely, Tanya Williamson, Christopher Sellar, Anil Abraham Joy, Kerry S. Courneya, Shirin M. Shallwani, Jacob Easaw, Sunita Ghosh, Harold Lau, Bruce Cameron, S. Nicole Culos-Reed

**Affiliations:** 1 Department of Physical Therapy, University of Alberta, 2-50 Corbett Hall, Edmonton, AB T6G 2G4, Canada; csellar@ualberta.ca (C.S.); sshallwa@ualberta.ca (S.M.S.); bruce.bcamron@gmail.com (B.C.); 2 Department of Oncology, University of Alberta, 11560 University Avenue NW, Edmonton, AB T6G 1Z2, Canada; anil.joy@ahs.ca (A.A.J.); jay.easaw@cancercare.ca (J.E.); sghosh1@ualberta.ca (S.G.); 3 Faculty of Kinesiology, University of Calgary, 2500 University Dr. NW, Calgary, AB T2N 1N4, Canada; willt@ucalgary.ca (T.W.); nculosre@ucalgary.ca (S.N.C.-R.); 4 Medical Oncology, Cross Cancer Institute, 11560 University Avenue NW, Edmonton, AB T6G 1Z2, Canada; 5 Faculty of Kinesiology, Sport, and Recreation, University of Alberta, 1-113 University Hall, Edmonton, AB T6G 2H9, Canada; kerry.courneya@ualberta.ca; 6 Research, Data and Analysis, Public Health Sciences Faculty, Henry Ford Hospital, 2799 West Grand Boulevard, Detroit, MI 48202, USA; 7 Department of Oncology, University of Calgary, 2500 University Dr. NW, Calgary, AB T2N 1N4, Canada; hlau@ucalgary.ca

**Keywords:** physical activity, exercise, implementation science, community-based participatory research, neoplasms, adult, prospective studies, treatment outcome

## Abstract

**Background/Objectives:** Despite recommendations to exercise, many individuals living with and beyond cancer do not achieve or sustain recommended physical activity levels because access to structured exercise support remains limited. We evaluated the real-world delivery of a scalable, community-based exercise model designed to address this gap. **Methods:** We conducted a hybrid effectiveness–implementation study of a 12-week supervised exercise intervention delivered in community settings across Alberta, Canada (in person or virtual). The primary outcome was the proportion of participants meeting physical activity guidelines at one year. Secondary outcomes included physical fitness and patient-reported outcomes. **Results:** Among 2570 participants, completion (90.6%) and attendance (77.1%) were high. At one year, the proportion meeting physical activity guidelines increased from 24.1% to 38.9% (*p* < 0.001), exceeding the prespecified target. At 12 weeks, participants demonstrated clinically meaningful gains in physical fitness, including sit-to-stand performance (+2.8 repetitions; 95% CI, 2.6, 3.9) and six-minute walk distance (+39.9 m; 95% CI, 36.9, 42.8), along with improvements in patient-reported outcomes. Improvements in fatigue and quality of life were smaller among participants receiving chemotherapy. Adverse events were rare (0.5%), with all serious events occurring among participants receiving active cancer treatment. **Conclusions:** This study demonstrates that structured supervision and behavioural support can be delivered through scalable, community-based models integrated with cancer care and may help patients achieve and sustain recommended physical activity levels. These findings support the translation of established exercise evidence into real-world models of care while highlighting the need for additional strategies to support long-term behaviour change.

## Simple Summary

This hybrid effectiveness–implementation study evaluated the real-world implementation of a 12-week community-based supervised exercise intervention (delivered virtually and in person) for individuals living with and beyond cancer. Among a cohort of 2570 participants, the intervention demonstrated high feasibility, achieving a 90.6% completion rate and a 77.1% attendance rate. The study met its primary outcome, demonstrating a statistically significant, sustained increase in the proportion of participants adhering to physical activity guidelines at the one-year follow-up (increasing from 24.1% at baseline to 38.9%, *p* < 0.001). Secondary outcome measures at 12 weeks showed clinically meaningful improvements in functional capacity including sit-to-stand performance and six-minute walk distance, as well as improvements in fatigue and quality of life. Adverse events were rare (0.5%), with all serious events occurring among participants receiving active cancer treatment. The findings support the translation of established exercise evidence into real-world models of care.

## 1. Introduction

Globally, cancer incidence continues to rise, with more than 20 million new cancers diagnosed annually [1]. Advances in cancer treatment have improved survival, creating a growing population of individuals living with and beyond cancer and shifting care priorities to optimizing physical function, psychosocial well-being, and quality of life throughout and after treatment [2]. Exercise is now recognized as an important component of cancer care [3]. A robust evidence base demonstrates that supervised exercise is safe and improves cardiorespiratory fitness, muscular strength, fatigue, and quality of life, while mitigating treatment-related toxicities and functional decline [4]. More recently, the landmark CHALLENGE trial, a large-scale randomized study of individuals with stage II and III colon cancer, demonstrated that structured exercise reduced mortality by 37% and recurrence or new cancers by 28%, with an 8-year overall absolute survival benefit of 7% [5]. This new evidence has led to heightened interest in implementing evidence-based exercise programs into routine cancer care [4,6,7].

Despite the proven benefits of exercise, fewer than one-third of individuals living with cancer meet recommended levels of physical activity [8]. Barriers such as treatment side effects, comorbidities, aging, and limited access to qualified exercise professionals restrict participation [9,10,11]. Even in the general population, physical activity levels remain consistently low (47%) [12,13], underscoring the broader challenges associated with changing physical activity behaviour [8]. The unresolved challenge is how to provide equitable, scalable access to structured cancer-specific exercise support capable of helping individuals achieve and sustain physical activity in routine care [14].

The Alberta Cancer Exercise (ACE) study was developed to address this gap through a clinic-to-community model delivering tailored, cancer-specific exercise across urban community sites in Alberta, Canada [15]. Guided by a hybrid effectiveness–implementation approach, ACE was conducted between 2017 and 2024, including adaptations to maintain access throughout and beyond the COVID-19 pandemic.

We report the primary effectiveness findings of ACE. We evaluated whether participation in the intervention would be associated with increased likelihood of meeting physical activity guidelines at one year, and assessed short-term changes in physical activity, physical fitness, and patient-reported outcomes following the 12-week intervention. We also examined outcomes according to treatment status and delivery context (in-person or virtual) and evaluated adherence and completion as indicators of intervention acceptability and feasibility.

## 2. Materials and Methods

### 2.1. Study Design and Participant Recruitment

ACE was designed to evaluate both participant outcomes (effectiveness) and delivery processes (implementation) [15]. The present analysis focuses on effectiveness outcomes including changes in physical activity, fitness, fatigue, and quality of life, while reporting key implementation adaptations relevant to intervention scalability. ACE opened in January 2017 and completed recruitment in February 2023. Details of the intervention design have been reported previously [15,16]. In brief, ACE integrates research and practice through an ongoing, province-wide partnership among universities, community fitness centres, and cancer care programs. Individuals with lived experience of cancer actively contributed to intervention design, delivery and interpretation of results to ensure a patient-centered approach. Participants were referred by healthcare providers or self-referred to ACE [17]. The Health Research Ethics Board of Alberta: Cancer Committee approved the study, and all participants provided written informed consent.

### 2.2. Setting and Intervention Delivery

Two hub sites, one at the University of Alberta (Edmonton) and the other at the University of Calgary (Calgary), were responsible for coordination, intervention oversight, training, and data collection. Each exercise session was delivered by qualified exercise professionals in partnership with local community or clinical facilities. Although the mode of delivery (in-person or virtual) was adapted as needed to ensure intervention and fitness testing continuity during the COVID-19 pandemic, the exercise prescription and data collection on key effectiveness outcomes remained consistent throughout the study period.

### 2.3. Eligibility Criteria

Eligible participants were adults (≥18 years) with a diagnosis of any cancer type or stage who were able to safely engage in mild-to-moderate exercise as determined by the respective hub Clinical Exercise Physiologist (CEP) or referring medical professional [17]. Individuals were eligible before, during, or within three years of treatment completion, including those receiving therapy for palliative intent or experiencing long-term or late treatment-related effects (e.g., lymphedema, radiation fibrosis syndrome, neurological deficits). Participants initially provided verbal consent for the collection of self-report data as part of the initial screening. Participants were excluded if they were not able to ambulate independently (with or without a gait aid) or transfer independently from sitting to standing. Written informed consent was obtained prior to full CEP screening and baseline fitness testing, and participants were triaged to a suitable community program [17], with higher-level supervision by a CEP or physical therapist for those with more complex medical presentations.

### 2.4. Exercise Intervention

ACE delivers a 12-week supervised exercise intervention offered three times annually (i.e., winter, spring, fall). Participants attend two supervised group sessions per week, delivered either in-person or virtually by ACE-trained qualified exercise professionals. The standardized exercise prescription consists of two supervised sessions per week, progressing in intensity from 3–4 (e.g., equivalent to brisk walking on a flat surface) to 4–5 metabolic equivalents (e.g., brisk uphill walking/stair climbing) over the 12-week intervention, incorporating aerobic, resistance, flexibility, and balance training components. Class formats included circuit-style and group personal-training options, with 8–15 participants per class. For virtual delivery, participants remained on camera for real-time supervision, and a second exercise professional monitored performance and safety, and provided technical support. Exercise-behaviour education included goal setting, activity monitoring, and strategies to increase daily physical activity.

### 2.5. Outcome Measures

Baseline and post-intervention assessments were conducted by CEPs using standardized procedures across all sites. Virtual assessments were implemented when in-person testing was not feasible. Consistent with the hybrid effectiveness–implementation design of ACE, both patient-reported and objective fitness measures were periodically refined to balance scientific rigor with real-world feasibility. Our initial battery of questionnaires was streamlined to reduce participant burden, and adaptations to fitness and body composition testing (e.g., virtual testing options, removal of less feasible measures) were implemented as the study enrollment increased and transitioned through pandemic restrictions (further details are provided below). Core outcome domains of physical activity, fitness, fatigue, and quality of life remained consistent throughout the study period to enable comparability across participants and cohorts (pre-, during and post-COVID).

Patient-reported outcomes were collected online at baseline, post-intervention, and one-year follow-up across all cohorts. Physical fitness and body composition tests were completed in person or virtually at baseline and post-intervention for all participants. One-year fitness testing was limited to participants from the tertiary centres (Edmonton and Calgary) and was discontinued after the initial grant funding period, which supported follow-up testing for the first 1000 participants.

The primary outcome was the proportion of participants meeting physical activity guidelines (≥150 min per week of moderate-to-vigorous physical activity) at the one-year follow-up as assessed by the modified Godin Leisure-Time Exercise Questionnaire [18]. Secondary effectiveness outcomes included continuous measures of physical activity, physical fitness, and patient-reported outcomes. Functional fitness was assessed using the 30 s sit-to-stand test. Aerobic capacity was assessed using the six-minute walk test (6MWT) for participants completing in-person assessments [19]. During periods when in-person testing was not feasible, virtual assessment included aerobic fitness assessed using the two-minute step test (2MST), a validated field test suitable for remote administration [20]. Where equipment and resources were available, muscular strength was assessed using handgrip dynamometry and through optional one-repetition maximum (1RM) testing. Secondary patient-reported outcomes included fatigue (FACIT-F) and quality of life (EQ5D-5L Visual Analogue Scale) [21].

Additional physiological and functional measures, including range of motion, plank endurance, and balance, as well as additional patient-reported outcomes, were collected as part of the broader implementation evaluation and are summarized in the Supplementary Materials. The severity of adverse events was graded according to the Common Terminology Criteria for Adverse Events (CTCAE) system [22]. Although a very low adverse event rate (<1%) was prespecified based on prior literature, the intervention was designed with the goal of minimizing serious adverse events [15].

### 2.6. Sample Size and Analyses

The initial recruitment target for the ACE study was 1000 participants; however, recruitment exceeded expectations during provincial implementation, and enrollment was expanded to 2500 participants. Based on prior feasibility data, a sample of 305 participants was estimated to provide 90% power (two-sided α = 0.01) to detect a 10% absolute increase in the proportion meeting physical activity guidelines at one year.

Clinical data, including diagnosis, treatment status, disease characteristics, and vital status, were verified through linkage with the Alberta Cancer Registry and electronic medical record review. Continuous variables are presented as mean ± standard deviation (SD) and categorical variables as frequencies and percentages. Baseline comparisons between subgroups were evaluated using analysis of variance (ANOVA) or χ^2^ tests, as appropriate. Within-participant changes in guideline attainment from baseline to one year were evaluated using McNemar’s test, and differences by treatment status using χ^2^ tests. Secondary analyses evaluated adjusted mean changes in physical activity, physical fitness, and patient-reported outcomes using generalized linear models, controlling for baseline values, biological sex, location, cancer type and stage, and age. Treatment status (chemotherapy, other treatment, or post-treatment) was the primary analytic factor, with delivery mode (in-person vs. virtual) examined as a secondary factor. Analyses by cancer type will be reported separately.

A sensitivity analysis excluding participants with breast cancer was conducted to account for potential overrepresentation and differing treatment trajectories. Exploratory subgroup analyses by sex and region (Edmonton, Calgary, North Zone, South Zone) were analyzed descriptively. Sites were categorized by operational delivery context into: (1) major metropolitan centers (Edmonton and Calgary; main hub sites with direct specialist staffing, high patient volume, and on-site implementation teams) and (2) regional urban centers (North Zone and South Zone; decentralized hub-and-spoke delivery model, lower population density, less direct implementation team contact).

Exploratory analyses also examined differences by intervention attendance (≥80% vs. <80%) for key outcomes (physical activity, sit-to-stand, fatigue and quality of life). These analyses were not prespecified. Statistical significance was defined as *p* < 0.05. Given the large sample size and exploratory nature of subgroup analyses, emphasis was placed on the direction and magnitude of effects rather than on statistical significance alone. Standardized mean differences (Cohen’s d) were calculated for key outcomes using adjusted mean changes and the baseline standard deviation of the analytic sample for each outcome. All analyses were performed using IBM SPSS Statistics (Version 29.0, Armonk, NY, USA).

To address missing one-year follow-up data (30.5% attrition), we performed Multiple Imputation by Chained Equations (MICE) using Fully Conditional Specification (FCS) under a Missing at Random assumption. The imputation model incorporated the one-year primary outcome, baseline physical activity status, demographic factors (age, sex, body mass index), and clinical characteristics (high vs. low-risk stage, cancer type, and location) to generate 10 imputed datasets. Binary logistic regression models were fitted to each dataset, and estimates were pooled to calculate pooled Odds Ratios (ORs) and 95% Confidence Intervals (95% CIs). To evaluate the robustness of our findings against potential missing data bias, we conducted two comparative sensitivity analyses alongside the pooled MICE model: (1) a complete-case analysis restricted to participants with available one-year follow-up data, and (2) a conservative non-responder sensitivity analysis (worst-case scenario) where all participants lost to follow-up at one year were assumed to be inactive (not meeting physical activity guidelines).

## 3. Results

**Figure 1:**
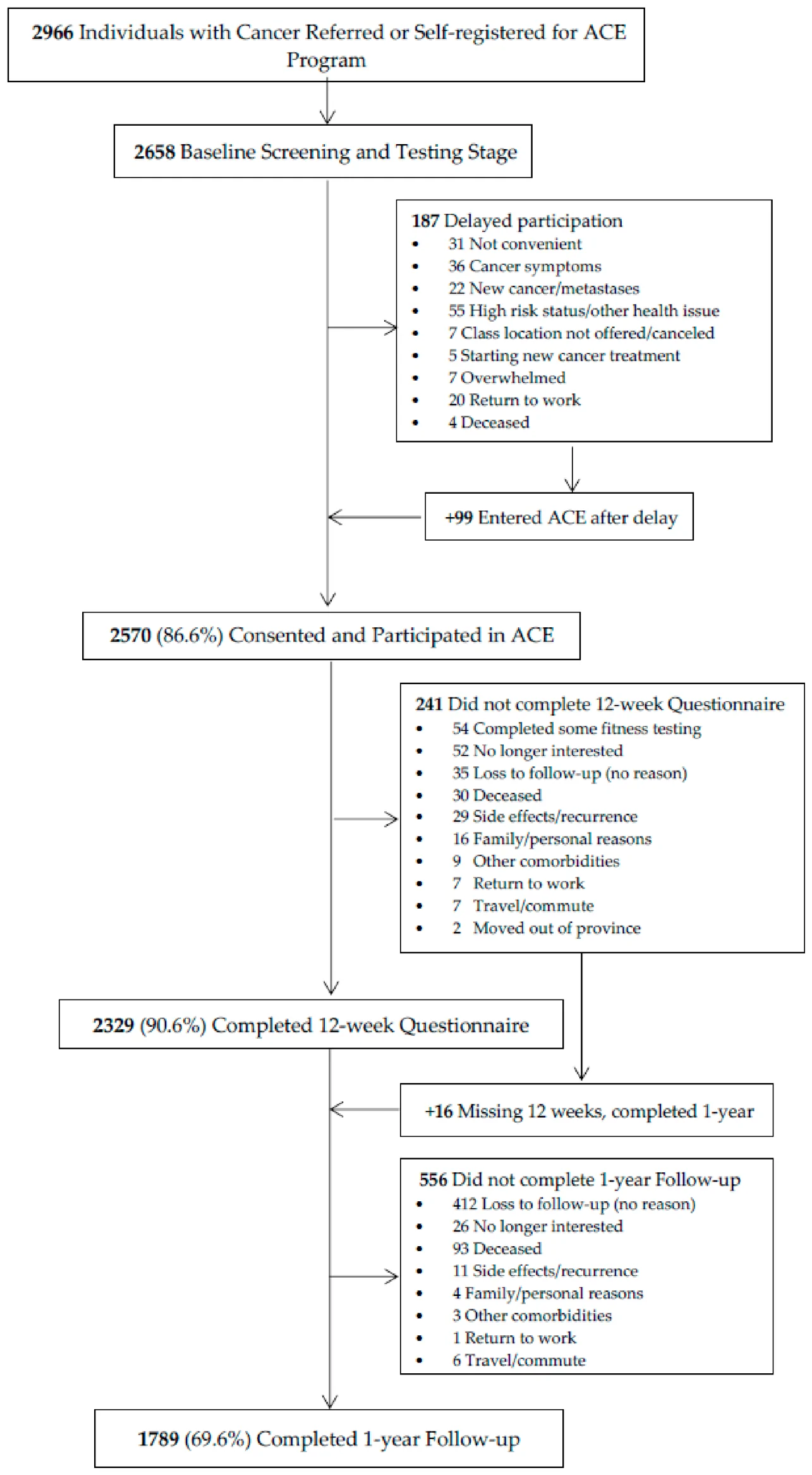
Study Flow Diagram.

A total of 2966 individuals with cancer were referred or self-referred to ACE, with 2570 consenting and participating in the study (Figure 1). Participants were predominantly female, with breast cancer the most common diagnosis (45.4%). At study entry, 27.8% of participants had high-risk or confirmed metastatic disease, and participants were enrolled across chemotherapy (n = 477), other active treatments (n = 777), and post-treatment groups (n = 1316) (Table 1).

### 3.1. Attendance and Completion Rates

Attendance during the 12-week intervention averaged 77.1%, with significantly lower attendance among participants receiving chemotherapy (72.5%) compared with those off treatment (77.8%) or receiving other treatments (78.6%) (*p* < 0.05). Intervention completion at 12 weeks was high (90.6%) and did not differ by treatment status (*p* > 0.05). Both attendance and 12-week completion rates were similar across in-person and virtual delivery modes (*p* > 0.05). At one-year follow-up, retention was higher among participants in the in-person intervention compared with virtual delivery (70.5% vs. 65.1%; *p* < 0.05).

### 3.2. Primary Outcome: Proportion Meeting Physical Activity Guideline Levels

The proportion of participants meeting physical activity guidelines increased significantly from baseline (24.1%) to one year (38.9%) (*p* < 0.001), exceeding the prespecified target of a 10% relative increase. In the primary pooled model using MICE adjusting for baseline physical activity, age, sex, body mass index, high-risk status, location, and cancer type, participants demonstrated 3.8 times higher odds of meeting physical activity guidelines at one year compared to baseline (*p* < 0.001). Findings were robust across sensitivity analyses, demonstrating similar estimates for both the complete-case analysis (restricted to follow-up completers) and the conservative non-responder analysis (Table 2).

**Figure 2:**
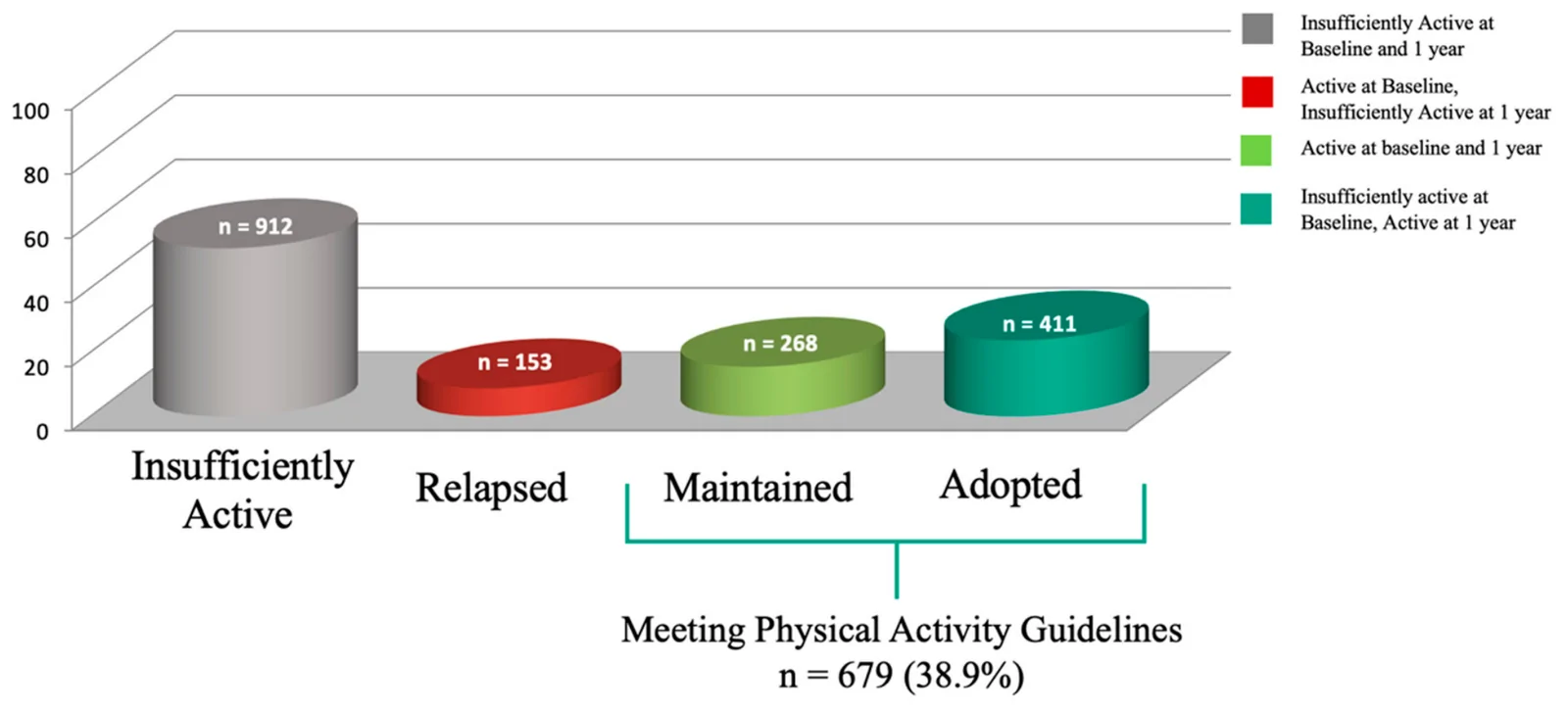
Distribution of Participants by Physical Activity Status at 1 year Relative to Baseline.

Participants were further categorized according to physical activity status at one year relative to baseline: 52.3% remained insufficiently active, 8.7% relapsed, 15.4% maintained guideline levels, and 23.6% adopted guideline-level physical activity (Figure 2). At one year, guideline attainment did not differ by treatment status (*p* = 0.828) and there was no significant interaction by cancer type. In adjusted models that included treatment status, baseline physical activity status was the strongest correlate of meeting physical activity guidelines at one year.

### 3.3. Secondary Outcomes: Physical Activity and Fitness Outcomes

**Figure 3:**
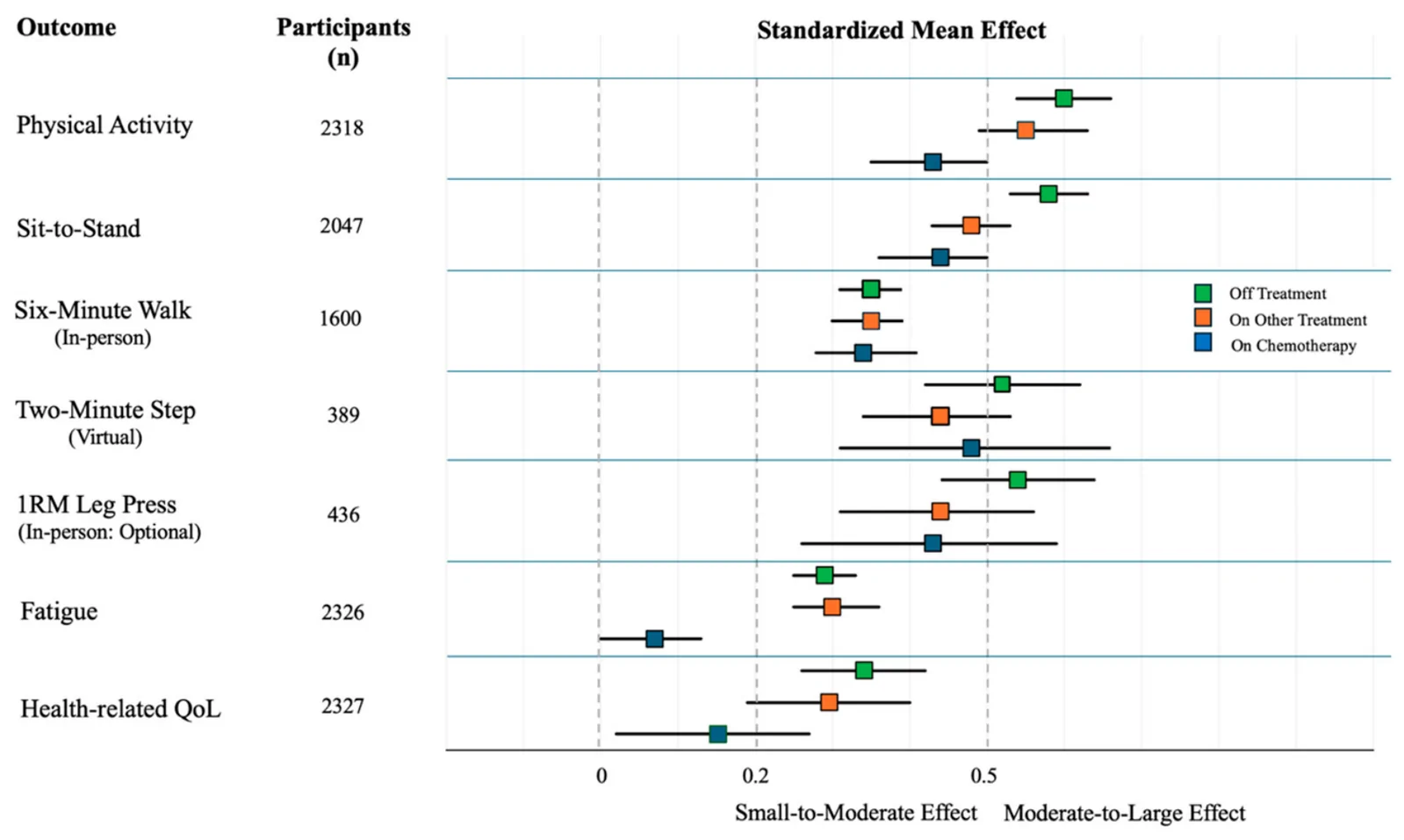
Standardized Mean Differences for Key Outcomes at 12 weeks across Treatment Groups.

At 12 weeks, moderate-to-vigorous physical activity increased by 74.9 min (95% CI: 69.4, 80.3) per week, with partial maintenance at one year (+50.9 min/week; 95% CI: 43.1, 58.7). Clinically meaningful improvements in physical fitness were observed at 12 weeks (Table 3). Sit-to-stand performance increased by 2.8 repetitions and six-minute walk test distance by 39.9 m, both exceeding established thresholds for clinical relevance. Handgrip strength improved but did not reach thresholds for clinical relevance (Table 3). Similar improvements were observed across treatment phases (Figure 3; Table 4) and delivery modes (Table 5), although modestly greater improvements in sit-to-stand and plank endurance performance were observed with in-person delivery.

#### Optional Strength Outcomes from Northern Sites

Among participants in northern sites who completed optional 1RM testing, both upper- and lower-body strength significantly increased. Overall, bench press strength increased by 4.9 kg and leg press strength by 15.5 kg, both exceeding the 10% threshold for clinically meaningful improvement (Table 3). Improvements did not differ significantly by treatment phase (*p* > 0.05) (Figure 3; Table 4).

### 3.4. Patient-Reported Outcomes

Symptoms and quality of life also improved following the intervention. Improvements in fatigue and health-related quality of life were clinically meaningful among participants who were off treatment or receiving other therapies but attenuated among those receiving chemotherapy (Figure 3). Improvements in fatigue met the lower bound of clinically meaningful thresholds (3–6 points) among participants who were off treatment (+3.20 points; standardized mean difference (SMD): 0.29) and receiving other treatments (+3.15 points; SMD: 0.30) but not among those receiving chemotherapy (+0.69 points; SMD: 0.07) (Table 4).

### 3.5. Effectiveness by Delivery Mode (In-Person and Virtual)

Outcomes were largely comparable between delivery modes (Table 5). Improvements in the 30 s sit-to-stand (+1.1; 95% CI: 0.7, 1.5) and plank endurance (+10.9; 95% CI: 4.9, 16.8) performance were modestly greater among participants in the in-person intervention compared with virtual delivery (*p* < 0.05). Changes in physical activity, fatigue (FACIT-F), and health-related QoL did not differ significantly between in-person and virtual formats (all *p* > 0.05). No significant interaction between treatment status and delivery mode was observed for any outcome (all *p* > 0.05).

### 3.6. Sensitivity, Subgroup and Exploratory Analyses

Sensitivity analyses excluding participants with breast cancer showed similar results to those observed in the full cohort (Supplementary Table S1). In subgroup analyses by biological sex, males demonstrated significantly larger absolute improvements in sit-to-stand repetitions, bench press, and grip strength, whereas females demonstrated significantly larger absolute improvements in six-minute walk test distance and sit-and-reach flexibility; no other outcomes differed significantly by sex (Supplementary Table S2). Intervention effects were broadly consistent across regions (Supplementary Table S3). Although larger improvements were observed in Calgary and the South for grip strength and in the South for shoulder range of motion compared with Edmonton, these differences were small and did not reach the minimal clinically important difference. Sit-and-reach flexibility improved across regions and met or exceeded the minimal clinically important difference, with larger gains observed in Calgary and the South. No significant differences in patient-reported outcomes were observed by sex or region. In exploratory analyses, participants with attendance ≥ 80% demonstrated significantly larger short-term improvements in physical activity, lower-body function (sit-to-stand), fatigue and quality of life at 12 weeks; however, no significant differences were observed for change in physical activity minutes at one year (Supplementary Table S4).

### 3.7. Adverse Events

Six serious adverse events (0.2%) and seven non-serious musculoskeletal adverse events (0.3%), occurring at or in association with the exercise intervention, were reported during the study period. All six serious adverse events occurred among participants receiving active cancer treatment, most involving individuals with advanced-stage or high-risk cancers. Events included three cardiac events (one CTCAE Grade 4; two CTCAE Grade 3) and one neurological event (CTCAE Grade 1) related to exercise participation, as well as one abdominal event (CTCAE Grade 3) and one fall-related event (CTCAE Grade 1), determined to be unrelated to exercise participation. One additional unrelated serious adverse event (CTCAE Grade 3: cardiac event) occurred during a routine medical procedure. All participants recovered without lasting sequelae.

## 4. Discussion

Among participants evaluable at one year, the proportion meeting physical activity guidelines increased from 24.1% at baseline to 38.9%, exceeding the prespecified 10% target [15]. In multivariable models accounting for baseline covariates and missing data through MICE, participants demonstrated 3.80 times higher odds of meeting guidelines at one year compared to baseline (OR: 3.80, 95% CI: 3.03, 4.90, *p* < 0.001). Importantly, the robustness of these findings was confirmed across sensitivity analyses, yielding consistent improvements under both complete-case (OR: 3.89) and conservative non-responder assumptions (OR: 3.35). These data indicate that for every six patients who participated in the ACE program, one additional patient was still meeting physical activity guidelines one year later (number needed to treat: 6). Moreover, participants increased their exercise from baseline by an average of 75 min at 12 weeks and 50 min at one year. These findings are clinically meaningful at a population level, as even modest increases in physical activity have been associated with reductions in chronic disease incidence and healthcare costs and lower cancer-specific mortality [15,23,24,25]. Despite increases in physical activity across cancer treatment groups, more than half of ACE participants (52.3%) remained insufficiently active at one year, underscoring the well-documented challenges of achieving sustained physical activity behaviour [26]. Interventions based primarily on information provision have consistently demonstrated only minimal or negligible effects on physical activity behaviour [8], suggesting that knowledge alone is insufficient to change behaviour [8]. More intensive and sustained behavioural support approaches may be required to promote long-term adherence [5]. Future work should evaluate strategies to enhance maintenance of physical activity following supervised intervention, including extended behavioural support, transition pathways to independent exercise, digital supports, and tailored approaches for individuals receiving active treatment.

Intervention completion was high (90.6%), and attendance exceeded the prespecified 70% target, supporting the acceptability and feasibility of ACE. These rates compare favourably with prior community-based exercise studies reporting lower adherence and higher dropout rates [27,28]. The supervised, group-based delivery model may have contributed to these outcomes by providing individualized support, accountability, and opportunities for social connection, factors previously associated with improved exercise participation and adherence [8]. ACE patient partner feedback further highlighted the perceived value of the intervention, with participants reporting improvements in physical abilities and mental outlook and, in some cases, describing the experience as life-changing. Together, these findings support the feasibility and acceptability of scalable, community-based exercise delivery.

Clinically meaningful improvements were observed across multiple fitness domains, including lower-body functional performance (sit-to-stand), functional exercise capacity (6MWT), aerobic endurance (2MST), and muscular strength (1RM measures). Benefits were generally smaller among individuals receiving chemotherapy, likely reflecting substantial symptom burden and treatment toxicities; however, meaningful improvements in physical fitness were still observed [29,30]. Findings are consistent with a large cohort study of individuals with breast cancer, where significant declines in self-reported physical health and functioning were found in participants receiving chemotherapy and/or endocrine therapy, with persistent long-term impairments in those who had received chemotherapy [31]. While modest advantages were observed for in-person participation, virtual delivery may improve accessibility for individuals facing geographic, treatment-related, or logistical barriers to participation. Moreover, exploratory analyses suggest greater short-term improvements in participants with ≥80% attendance, consistent with a dose–response pattern [32]. Importantly, the ACE study demonstrates how fitness testing and intervention delivery can be adapted to virtual formats, broadening the reach and scalability of supervised community-based exercise.

Clinically meaningful improvements in fatigue and quality of life were observed among participants who were off treatment or receiving other therapies, whereas individuals undergoing chemotherapy experienced smaller effects. Although prior reviews have reported benefits of exercise for fatigue and quality of life [33,34,35], the ACE findings are consistent with a recent systematic review reporting modest and non-significant effects of low-to-moderate-intensity exercise on fatigue in individuals undergoing chemotherapy [36]. Cancer-related fatigue is multifactorial, encompassing biological, physiological, and psychosocial processes, and exercise alone may be insufficient to fully address treatment-related effects such as cytotoxicity, nutritional deficits, and sleep disturbance [37]. Given the substantial symptom burden associated with chemotherapy, these findings suggest that exercise may help attenuate symptom worsening while still providing important benefits, particularly for physical fitness. In contrast, individuals receiving other treatments, including hormonal and targeted therapies, may experience fewer treatment-related side effects [31,38], facilitating greater participation and improvements in both objective and patient-reported outcomes. Collectively, these findings underscore the importance of tailoring exercise interventions to treatment status and providing additional monitoring and support for individuals undergoing chemotherapy [39]. Multimodal approaches that integrate exercise with symptom management strategies may further optimize outcomes, particularly for individuals receiving chemotherapy treatment.

Our subgroup analysis across urban sites demonstrates the scalability of the intervention. Importantly, physical activity guideline attainment remained high across both major metropolitan centers and regional urban zones, despite the latter having less direct team contact. These findings offer important guidance for other international jurisdictions and health systems seeking to scale exercise oncology programs beyond specialized tertiary cancer centers into underserved regional and lower-density communities where cancer-specific exercise implementation support may be limited [40,41].

### 4.1. Adverse Events

The low rate of adverse events supports the overall safety of supervised exercise in community settings, although risks cannot be completely eliminated, particularly among individuals receiving active treatment [42,43]. These findings reinforce the importance of appropriate screening, monitoring, and delivery by qualified exercise professionals when implementing scalable exercise models for individuals living with and beyond cancer [42,43,44,45,46].

### 4.2. Strengths and Limitations

A key strength of this study is the demonstration that a structured exercise intervention can be implemented at scale in community settings. Co-designed with patient partners, ACE community-based delivery and flexible intervention formats may facilitate broader reach and uptake, particularly given the limited capacity of healthcare systems to deliver exercise services directly [14,45]. An additional strength is the verification of clinical and treatment data through cancer registry linkage and medical record abstraction, enhancing data accuracy and addressing a common limitation of implementation studies that rely on self-reported or unverified data. Limitations include the lack of a control group, the predominance of female participants and individuals with breast cancer, and the variability introduced by the transition to virtual delivery during the COVID-19 pandemic.

## 5. Conclusions

In this hybrid effectiveness–implementation study, the ACE intervention was associated with clinically meaningful improvements in physical activity, physical fitness, and patient-reported outcomes, with sustained increases in guideline-level physical activity at one year. High completion and adherence further support the feasibility of scalable, community-based exercise for individuals living with and beyond cancer. Although many participants remained insufficiently active, these findings support the integration of structured exercise support into cancer care as the field expands from efficacy to implementation.

### Research, Educational and Policy Implications

Recommendations for future implementation research include evaluating strategies to enhance long-term physical activity maintenance post-intervention such as extended behavioral support, use of digital tools, tailored pathways for individuals during active cancer treatment, and hybrid delivery models (in-person and virtual) adapted from lessons learned during the COVID-19 pandemic. Moreover, additional research in underrepresented populations, beyond breast cancer, is warranted.

Health system integration requires targeted educational initiatives for oncology providers to promote referral to exercise programs, and to facilitate exercise counseling as part of standard care pathways, alongside training for exercise professionals to design and deliver cancer-specific programming.

Finally, sustainable scaling of programming requires policy that recognizes exercise as an essential supportive cancer care service, ensuring dedicated healthcare funding, reimbursement models, and strategic partnerships with community fitness settings to reduce geographic and access barriers for individuals with cancer.

## Figures and Tables

**Table 1:** Participant Demographic and Clinical Characteristics Overall and by Treatment Status.

Demographic Characteristic	Total Cohort (N = 2570)		Completed Cancer Treatment (n = 1316)		On Other Treatment (n = 777)		On Chemotherapy Treatment (n = 477)	
	Mean/N	SD/%	Mean/N	SD/%	Mean/N	SD/%	Mean/N	SD/%
A ge at diagnosis, yrs	57.8	12.0	58.6	12.1	57.3	11.5	56.2	12.4
17 to 39 years	190	7.4%	95	7.2%	48	6.2%	47	9.9%
40 to 64 years	1582	61.6%	762	57.9%	502	64.6%	318	66.7%
65+ years	798	31.1%	459	34.9%	227	29.2%	112	23.5% *
Biological Sex								
Female	1832	71.3%	863	65.6%	632	81.3%	337	70.6%
Male	738	28.7%	453	34.4%	145	18.7%	140	29.4%
Primary Cancer Type								
Breast	1167	45.4%	434	33.0%	550	70.8%	183	38.4%
Hematologic	355	13.8%	191	14.5%	52	6.7%	112	23.5%
Genitourinary	241	9.4%	143	10.9%	85	10.9%	13	2.7%
Gastrointestinal	231	9.0%	152	11.6%	10	1.3%	69	14.5%
Head and Neck	159	6.2%	148	11.2%	8	1.0%	3	0.6%
Gynecologic	152	5.9%	98	7.4%	20	2.6%	34	7.1%
Neurologic	93	3.6%	48	3.6%	2	0.3%	43	9.0%
Lung	90	3.5%	43	3.3%	32	4.1%	15	3.1%
Other	82	3.2%	59	4.5%	18	2.3%	5	1.0%
Metastatic or High Risk	714	27.8%	333	46.6%	162	22.7%	219	30.7%
Alberta Region								
Calgary	1149	44.7%	590	44.8%	339	43.6%	220	46.1%
Edmonton	1122	43.7%	569	43.2%	347	44.7%	206	43.2%
South Zone	122	4.7%	61	4.6%	39	5.0%	22	4.6%
North Zone	177	6.9%	96	7.3%	52	6.7%	29	6.1%

* significantly different *p* < 0.05.

**Table 2:** Multivariable Logistic Regression Models for Physical Activity Guideline Attainment at One Year.

Model/Analysis Method	Missing Data Assumption	Sample Size	Adjusted OR [95% CI] *	*p* Value
Imputed Model (MICE)	Missing at Random	2570	3.8 [3.0, 4.9]	*p * < 0.001
Complete-Case Analysis	Excludes missing data	1744	3.9 [3.1, 4.9]	*p * < 0.001
Worst-Case Non-responder Analysis	Missing = assigned as inactive classification	2570	3.6 [2.8, 4.1]	*p * < 0.001

* Adjusting for baseline physical activity, age, sex, body mass index, location, stage (high/low risk), and cancer type.

**Table 3:** Overall Results Post-Intervention (12 weeks).

Outcome	Metric	Baseline Mean (SD) (N = 2570)	12 Weeks Mean (SD) (N = 2329)	Sample Size	Adjusted Mean Change [95% CI]	*p*-Value
Physical Activity Minutes/Week	Minutes	85.4 (135.8)	159.2 (146.0)	2317	+74.9 [69.4, 80.3]	*p * < 0.001 ^†^
30 s Sit-to-Stand	Repetitions	14.2 (5.3)	17.1 (6.2)	2047	+2.8 [2.6, 2.9]	*p * < 0.001 ^†^
Six-minute Walk Test (In-person only)	Metres	539.1 (110.0)	580.4 (107.2)	1600	+39.6 [36.6, 42.5]	*p * < 0.001 ^†^
Two-minute Step Test (Virtual Only)	Repetitions	75.6 (22.0)	89.0 (21.5)	389	+12.0 [10.4, 13.6]	*p * < 0.001 ^†^
Combined Grip Strength (In-person only)	Kilograms	63.3 (18.9)	65.3 (19.3)	1603	+1.9 [1.5, 2.2]	*p * < 0.001
Shoulder Flexion (Right)	Degrees	148.2 (13.6)	150.3 (12.8)	2064	+1.9 [1.5, 2.3]	*p * < 0.001
Shoulder Flexion (Left)	Degrees	146.9 (13.7)	149.1 (12.7)	2062	+2.1 [1.8, 2.5]	*p * < 0.001
Average One-leg Balance	Seconds	27.2 (16.0)	31.4 (15.1)	1941	+3.9 [3.5, 4.3]	*p * < 0.001
Bench Press (1RM: Optional In-person)	Kilograms	35.2 (16.6)	40.7 (18.1)	500	+4.9 [4.2, 5.6]	*p * < 0.001 ^†^
Leg Press (1RM: Optional In-person)	kilograms	77.7 (33.3)	93.2 (40.4)	436	+15.5 [13.4, 17.7]	*p * < 0.001 ^†^
Plank Endurance Test (Optional)	Seconds	47.7 (38.4)	75.1 (52.4)	745	+27.0 [24.8, 29.2]	*p * = 0.003 ^†^
Lower Body Flexibility (Optional)	Centimetres	18.9 (14.0)	21.4 (13.9)	1753	+2.8 [2.5, 3.0]	*p * < 0.001
ESAS Total Score *	Score: 0–90	17.1 (12.9)	16.0 (12.4)	2319	−1.1 [0.7, 1.4]	*p * < 0.001
FACT-G	Score: 0–160	75.8 (15.1)	78.1 (14.9)	2317	+1.6 [1.3, 2.0]	*p * < 0.001
FACIT-Fatigue Scale	Score: 0–52	34.8 (10.9)	37.9 (10.2)	2325	+2.7 [2.4, 3.0]	*p * < 0.001
FACT: Trial Outcome Index	Score: 0–108	73.0 (19.2)	78.1 (18.5)	2321	+4.4 [3.9, 4.9]	*p * < 0.001
EQ-5D-5L Visual Analogue Scale	Score: 0–100	65.7 (17.8)	71.3 (17.0)	2327	+5.1 [4.5, 5.6]	*p * < 0.001

Adjusting for baseline value, age, sex, location, stage, and cancer type; * Lower scores: less symptom burden; ^†^ Meeting a priori targets based on minimum clinically important difference.

**Table 4:** Analyses by Treatment Status.

Outcome	Metric	Baseline Assessment T0 Mean (SD)	12-Week Post-Intervention T1 Mean (SD)	Sample Size T0 to T1	Adjusted Mean Difference: T0 to T1 Mean Change [95% CI]	Adjusted Difference vs. on Chemotherapy: T0 to T1 Mean Difference [95% CI]	*p*-Value
Physical Activity Off Treatment On Other Treatment On Chemotherapy	Minutes	85.3 (129.6) 93.1 (146.3) 79.5 (131.0)	160.7 (141.9) 171.0 (156.8) 135.0 (135.9)	1189 708 420	78.7 [71.0, 86.5] 81.3 [71.3, 91.3] 53.2 [40.2, 66.2]	+25.5 [6.8, 44.3] +28.1 [8.2, 48.0] Reference	*p* = 0.003 *p* = 0.002
30 s Sit-to-Stand Off Treatment On Other Treatment On Chemotherapy	Repetitions	14.5 (5.3) 14.4 (5.1) 14.2 (5.1)	17.4 (6.2) 17.1 (6.0) 16.7 (6.1)	1043 642 362	2.9 [2.7, 3.2] 2.6 [2.3, 2.9] 2.4 [2.0, 2.8]	+0.5 [−0.04, 1.1] +0.2 [−0.4, 0.8] Reference	*p* = 0.074 *p* = 1.000
Six-minute Walk Test (In-person only) Off Treatment On Other Treatment On Chemotherapy	Metres	538.3 (106.4) 545.4 (104.2) 543.2 (113.6)	578.0 (107.7) 584.6 (105.2) 582.9 (106.9)	841 485 274	39.3 [35.1, 43.5] 39.6 [34.1, 45.1] 40.3 [33.1, 47.6]	−1.0 [−11.4, 9.3] −0.8 [−11.8, 10.3] Reference	*p* = 1.000 *p* = 1.000
Two-minute Step Test (Virtual Only) Off Treatment On Other Treatment On Chemotherapy	Repetitions	76.6 (21.7) 77.5 (21.6) 77.2 (23.7)	88.9 (20.7) 90.3 (22.3) 87.1 (22.2)	183 132 74	12.0 [9.6, 14.4] 13.1 [10.3, 15.9] 10.2 [6.5, 14.0]	+2.3 [−3.0, 7.6] +3.0 [−2.6, 8.5] Reference	*p* = 1.000 *p* = 0.690
Combined Grip Strength (In-person only) Off Treatment On Other Treatment On Chemotherapy	Kilograms	64.9 (19.5) 60.5 (17.4) 64.1 (17.8)	67.0 (20.3) 62.4 (17.0) 65.3 (19.1)	841 487 275	2.1 [1.6, 2.7] 1.8 [1.2, 2.5] 1.1 [0.2, 2.0]	+1.0 [−0.2, 2.3] +0.7 [−0.6, 2.1] Reference	*p* = 0.155 *p* = 1.000
Shoulder Flexion (Right) Off Treatment On Other Treatment On Chemotherapy	Degrees	147.1 (14.0) 149.7 (12.6) 149.6 (12.8)	149.6 (13.2) 151.0 (13.2) 151.2 (13.2)	1050 651 363	2.1 [1.6, 2.6] 1.6 [0.9, 2.3] 1.9 [1.0, 2.8]	+0.2 [−1.1, 1.5] −0.3 [−1.7, 1.1] Reference	*p* = 1.000 *p* = 1.000
Shoulder Flexion (Left) Off Treatment On Other Treatment On Chemotherapy	Degrees	146.4 (14.1) 147.7 (12.5) 147.9 (13.6)	148.7 (12.8) 149.7 (12.0) 149.8 (13.5)	1051 648 363	2.2 [1.6, 2.7] 2.1 [1.5, 2.8] 2.0 [1.1, 2.9]	+0.1 [−1.1, 1.4] +0.1 [−1.2, 1.5] Reference	*p* = 1.000 *p* = 1.000
Average One-leg Balance Off Treatment On Other Treatment On Chemotherapy	Seconds	26.7 (16.0) 28.6 (15.6) 30.0 (15.9)	30.6 (15.4) 33.0 (14.4) 32.2 (15.1)	993 600 348	3.8 [3.2, 4.3] 4.5 [3.7, 5.2] 3.4 [2.4, 4.4]	+0.4 [−1.0, 1.8] +1.1 [−0.4, 2.6] Reference	*p* = 1.000 *p* = 0.250
Bench Press (1RM: Optional In-person) Off Treatment On Other Treatment On Chemotherapy	Kilograms	37.8 (16.6) 33.6 (14.8) 33.8 (18.1)	42.7 (18.2) 38.7 (16.8) 38.3 (19.6)	272 150 78	4.7 [3.8, 5.7] 5.6 [4.2, 6.9] 4.3 [2.3, 6.0]	+0.4 [−2.2, 3.0] +1.3 [−1.5, 4.1] Reference	*p* = 1.000 *p* = 0.803
Leg Press (1RM: Optional In-person) Off Treatment On Other Treatment On Chemotherapy	Kilograms	78.7 (30.8) 78.9 (32.7) 75.7 (33.2)	95.6 (41.6) 92.8 (40.5) 90.0 (39.4)	238 130 68	16.8 [13.8, 19.8] 13.7 [9.6, 17.8] 14.8 [9.1, 20.4]	+2.0 [−5.9, 10.0] −1.0 [−9.6, 7.5] Reference	*p* = 1.000 *p* = 1.000
Plank Endurance Test (Optional) Off Treatment On Other Treatment On Chemotherapy	Seconds	50.8 (43.8) 45.4 (34.1) 50.5 (34.8)	80.6 (58.3) 70.8 (47.6) 72.7 (47.6)	366 251 128	29.5 [26.2, 32.7] 26.0 [22.1, 29.8] 22.0 [16.4, 27.2]	+7.7 [−0.1, 15.5] +4.2 [−3.9, 12.3] Reference	*p* = 0.056 *p* = 0.652
Lower Body Flexibility (Optional) Off Treatment On Other Treatment On Chemotherapy	Centimetres	18.2 (14.3) 18.8 (14.3) 20.6 (13.2)	21.0 (14.1) 21.8 (13.8) 22.7 (13.2)	908 541 304	2.9 [2.5, 3.3] 2.8 [2.3, 3.3] 2.2 [1.5, 2.9]	+0.7 [−0.2, 1.6] +0.6 [−0.4, 1.6] Reference	*p* = 0.200 *p* = 0.412
ESAS Total Score Off Treatment On Other Treatment On Chemotherapy	Score: 0–90	17.1 (12.6) 17.5 (12.9) 16.3 (12.3)	15.8 (12.4) 16.0 (12.6) 16.5 (12.2)	1190 710 419	1.2 [0.7, 1.7] 1.4 [0.7, 2.0] 0.3 [−0.6, 1.2]	−0.9 [−0.4, 2.2] −1.1 [−0.3, 2.5] Reference	*p* < 0.264 *p* < 0.175
FACT-G (0–160) Off Treatment On Other Treatment On Chemotherapy	Score: 0–160	77.0 (15.4) 76.0 (14.6) 75.5 (13.3)	78.8 (15.2) 78.2 (147) 75.7 (13.7)	1187 709 421	1.9 [1.4, 2.5] 2.1 [1.4, 2.8] 0.1 [−0.9, 1.1]	+1.8 [0.5, 3.1] +1.9 [0.5, 3.3] Reference	*p* < 0.004 *p* < 0.003
FACIT-Fatigue Scale Off Treatment On Other Treatment On Chemotherapy	Score: 0–52	35.4 (11.0) 35.1 (10.4) 34.4 (10.4)	38.4 (10.2) 38.3 (9.8) 35.4 (10.6)	1192 711 422	3.2 [2.7, 3.6] 3.2 [2.6, 3.7] 0.7 [−0.0, 1.5]	+2.4 [1.4, 3.5] +2.4 [1.3, 3.6] Reference	*p* < 0.001 *p* < 0.001
FACT: Trial Outcome Index Off Treatment On Other Treatment On Chemotherapy	Score: 0–108	74.5 (19.6) 73.6 (18.1) 71.6 (17.9)	79.4 (18.6) 78.9 (17.7) 73.0 (19.0)	1190 710 421	5.1 [4.4, 5.9] 5.2 [4.2, 6.1] 1.0 [−0.3, 2.2]	+4.2 [2.4, 6.0] +4.2 [2.3, 6.1] Reference	*p* < 0.001 *p* < 0.001
EQ-5D-5L Visual Analogue Scale Off Treatment On Other Treatment On Chemotherapy	Score: 0–100	66.3 (17.2) 67.3 (17.2) 64.5 (18.0)	72.1 (16.8) 72.0 (16.4) 68.0 (18.2)	1193 711 423	5.8 [5.0, 6.6] 5.1 [4.1, 6.1] 3.0 [1.6, 4.3]	+2.8 [0.9, 4.8] +2.1 [0.1, 4.2] Reference	*p* = 0.002 *p* = 0.041

Adjusted differences were estimated from models with chemotherapy as the reference group and adjusted for baseline outcome values and covariates of age, sex, and location.

**Table 5:** Analysis by Delivery Mode.

Outcome		T0 Baseline Assessment Mean (SD)	T1 12-Week Post-Intervention Mean (SD)	Sample Size T0 to T1	Adjusted Within Subgroup Mean Difference: T0 to T1 Mean Change [95% CI]	Adjusted Between Subgroup Mean Difference: T0 to T1 Mean Difference [95% CI]	*p*-Value
Physical Activity In-person Virtual	Minutes	84.8 (134.3) 87.6 (141.7)	159.4 (143.5) 158.2 (156.0)	1854 463	75.4 [69.3, 81.4] 72.9 [60.7, 85.1]	+2.6 [−11.0, 16.2]	*p* = 0.720
30 s Sit-to-Stand In-person Virtual	Repetitions	14.6 (5.3) 12.8 (4.8)	17.6 (6.3) 15.1 (5.3)	1614 433	3.0 [2.8, 3.2] 1.9 [1.5, 2.2]	+1.1 [0.7, 1.5]	*p* < 0.001
Average One-leg Balance In-person Virtual	Seconds	26.4 (16.0) 30.2 (15.6)	30.8 (15.3) 33.6 (14.4)	1505 436	4.0 [3.5, 4.4] 3.7 [2.8, 4.6]	+0.3 [−0.7, 1.3]	*p* = 0.589
Plank Endurance Test (Optional) In-person Virtual	Seconds	47.8 (39.2) 47.5 (33.8)	76.9 (54.8) 65.8 (36.2)	623 122	28.8 [26.4, 31.2] 17.8 [12.3, 23.2]	+11.0 [5.0, 16.9]	*p* < 0.001
ESAS Total Score In-person Virtual	Score: 0–90	17.5 (13.0) 17.9 (12.6)	15.9 (12.3) 16.5 (12.6)	1855 464	1.1 [0.7, 1.5] 1.0 [0.2, 1.8]	+0.1 [−0.9, 1.0]	*p* = 0.857
FACT-G (0–160) In-person Virtual	Score: 0–160	76.2 (15.1) 74.1 (14.8)	78.4 (14.8) 76.8 (15.0)	1851 466	1.5 [1.1, 2.0] 2.1 [1.2, 2.9]	+0.5 [−0.4, 1.5]	*p* = 0.285
FACIT-Fatigue Scale In-person Virtual	Score: 0–52	35.0 (10.9) 34.1 (10.9)	37.9 (10.2) 37.7 (10.2)	1859 466	2.6 [2.3, 3.0] 3.2 [2.5, 3.9]	+0.6 [−0.2, 1.3]	*p* = 0.158
FACT: Trial Outcome Index In-person Virtual	Score: 0–108	73.5 (19.2) 71.0 (19.1)	78.4 (18.4) 77.0 (19.0)	1855 466	4.2 [3.6, 4.8] 5.2 [4.0, 6.4]	+1.0 [−0.3, 2.3]	*p* = 0.143
EQ-5D-5L Visual Analogue Scale In-person Virtual	Score: 0–100	66.0 (17.8) 64.5 (17.9)	71.7 (16.8) 70.0 (17.8)	1861 466	5.1 [4.5, 5.8] 4.8 [3.5, 6.1]	+0.3 [−1.1, 1.7]	*p* = 0.658

## Data Availability

The data supporting the findings of this implementation study are available from the corresponding author upon reasonable request. The complete database is not currently publicly available to protect ongoing, multi-phase research initiatives from this project and to maintain the privacy of participating community locations, healthcare institutes and personnel.

## Supplementary Materials

The following supporting information can be downloaded at https://www.mdpi.com/article/10.3390/cancers18162641/s1, Supplementary Table S1: Sensitivity Analyses Excluding Breast Cancer Group; Supplementary Table S2: Subgroup Analyses by Biological Sex; Supplementary Table S3: Subgroup Analyses by Region; Supplementary Table S4: Key Outcomes by Adherence.

## Abbreviations

The following abbreviations are used in this manuscript:

ACE	Alberta Cancer Exercise
CEP	Clinical Exercise Physiologist
6MWT	Six-minute walk test
2MST	Two-minute step test
1RM	One-repetition maximum test
