# Supplementary material for: Scalable Community-Based Exercise to Support Physical Activity in Cancer Care: A Hybrid Effectiveness-Implementation Study
Source: Cancers (Basel). 2026 Aug 16;18(16):2641. doi: 10.3390/cancers18162641 (PMC13511498; doi:10.3390/cancers18162641)
Supplement: Supplementary file 1 [file cancers-18-02641-s001.zip › cancers-4428256-supplementary.pdf]

Supplementary Table S1. Sensitivity Analyses Excluding Breast Cancer Group

Outcome	Sample Size	Full Cohort: Adjusted Mean Difference* [95% CI]	Sample Size	Non-Breast Cancer Group: Adjusted Mean Difference* [95% CI]
Physical Activity, minutes	2317	+74.9 [69.4, 80.3]	1256	+70.5 [62.1, 79.0]
30 second Sit-to-Stand, repetitions	2047	+2.8 [2.6, 3.9]	1105	+2.6 [2.4, 2.9]
Six-minute Walk Test, m (In-person only)	1600	+39.7 [36.7, 42.7]	891	+38.6 [34.0, 43.2]
Two-minute Step Test (Virtual Only)	389	+12.0 [10.4, 13.6]	190	+12.3 [9.7, 15.0]
Combined Grip Strength, kg (In-person only)	1603	+1.9 [1.5, 2.3]	889	+1.8 [1.2, 2.3]
Shoulder Flexion, degrees (Right)	2064	+1.9 [1.5, 2.3]	1109	+2.0 [1.4, 2.6]
Shoulder Flexion, degrees (Left)	2062	+2.1 [1.8, 2.5]	1108	+2.1 [1.5, 2.7]
Average One-leg Balance, seconds	1941	+4.0 [3.6, 4.4]	1049	+3.3 [2.7, 4.0]
Bench Press, kg (1RM: Optional In-person)	500	+4.8 [4.1, 5.6]	307	+5.0 [4.0, 6.1]
Leg Press, kg (1RM: Optional In-person)	436	+15.3 [13.1, 17.6]	262	+16.5 [13.3, 19.8]
Plank Endurance Test (Optional)	745	+27.1 [24.9, 29.3]	402	+25.6 [22.1, 29.0]
Lower Body Flexibility, cm (Optional)	1753	+2.8 [2.5, 3.0]	952	+2.7 [2.2, 3.1]
ESAS-r Total Score	2319	+1.2 [0.7, 1.5]	1257	+0.6 [0.1, 1.2]
FACT-G (0-160)	2317	+1.7 [1.3, 2.1]	1256	+1.2 [0.6, 1.8]
FACIT-Fatigue Scale	2325	+2.7 [2.4, 3.1]	1260	+2.5 [2.0, 2.9]
FACT: Trial Outcome Index	2321	+4.4 [3.9, 5.0]	1257	+3.9 [3.1, 4.7]
EQ-5D-5L Visual Analogue Scale	2327	+5.1 [4.5, 5.7]	1261	+4.5 [3.6, 5.3]

**Controlling for baseline value, age, sex, location, stage/risk status, treatment status.*

Supplementary Table S2. Subgroup Analyses by Biological Sex

Outcome	Sample Size	Adjusted Mean Difference T0-T1 [95% CI]	Adjusted between subgroup mean difference: T0 to T1 Mean Difference [95% CI]
Physical Activity, minutes			
Females	1666	73.2 [67.2, 90.9]	
Males	651	79.1 [66.4, 80.2]	5.9 [-8.8, 20.5]
30 second Sit-to-Stand, repetitions			
Females	1471	2.6 [2.4, 2.8]	
Males	576	3.2 [2.9, 3.6]	0.7 [0.2, 0.7]; p =0.004
Six-minute Walk Test, m (In-person only)			
Females	1107	46.2 [40.0, 52.4]	9.6 [1.7, 17.5]; p = 0.017
Males	493	36.6 [37.9, 48.6]	
Two-minute Step Test (Virtual Only)			
Females	312	12.1 [10.3, 13.9]	0.5 [-4.1, 4.1]
Males	77	11.6 [7.5, 15.8]	
Shoulder Flexion, degrees (Right)			
Females	1487	2.1 [1.7, 2.6]	0.9 [-0.2, 1.9]
Males	577	1.3 [0.4, 2.1]	
Shoulder Flexion, degrees (Left)			
Females	1487	2.3 [1.8, 2.7]	0.5 [-0.5, 1.5]
Males	575	1.8 [0.9, 2.6]	
Average One-leg Balance, seconds			
Females	1402	4.2 [3.3, 5.2]	0.5 [-0.7, 1.6]
Males	539	3.8 [3.3, 4.3]	
Combined Grip Strength, kg (In-person only)			
Females	1114	0.4 [-0.1, 1.0]	
Males	490	5.2 [4.2, 6.1]	4.8 [3.5, 6.0]; p < 0.001
Bench Press, kg (1RM: Optional)			
Females	312	2.8 [1.6, 3.9]	
Males	188	8.3 [6.7, 10.0]	5.6 [3.1, 7.9]; p < 0.001
Leg Press, kg (1RM: Optional)			
Females	274	13.5 [10.3, 16.7]	
Males	162	18.9 [14.4, 23.5]	3.3 [-1.6, 8.3]
Plank Endurance Test, secs (Optional)			
Females	522	25.8 [22.9, 28.7]	
Males	223	29.6 [22.9, 34.5]	3.8 [-2.3, 10.0]
Lower Body Flexibility, cm (Optional)			
Females	1237	3.0 [2.7, 3.4]	0.9 [0.2, 1.7]; p = 0.014
Males	516	2.1 [1.5, 2.7]	

ESAS-r Total Score			
Females	1667	1.1 [0.6, 1.6]	0.1 [-0.9, 1.1]
Males	652	1.0 [0.2, 1.8]	
FACT-G (0-160)			
Females	1665	1.7 [1.3, 2.2]	+0.3 [-0.7, 1.3]
Males	652	1.4 [0.6, 2.3]	
FACIT-Fatigue Scale			
Females	1671	2.8 [2.5, 3.2]	+0.4 [-0.4, 1.3]
Males	654	2.4 [1.8, 3.1]	
FACT: Trial Outcome Index			
Females	1669	4.5 [3.9, 5.2]	+0.5 [-0.9, 1.9]
Males	652	4.0 [2.9, 5.2]	
EQ-5D-5L Visual Analogue Scale			
Females	1672	5.3 [4.6, 6.0]	
Males	655	4.5 [3.4, 5.5]	+0.8 [-0.8, 2.3]

**Controlling for baseline value, age, location, stage/risk status, treatment status, cancer type; SD: standard deviation; CI: confidence interval; 1-RM: one-repetition maximum; ESAS: Edmonton Symptom Assessment System-Revised; **significant between subgroup change p < 0.05*

Supplementary Table S3. Subgroup Analyses by Region

Outcome	Location	Sample Size T0 to T1	Adjusted mean difference*: T0 to T1 [95% CI]
Physical Activity, minutes	Edmonton	1015	69.2 [61.0, 77.4]
	Calgary	1034	79.4 [71.3, 87.6]
	North Zone	157	67.9 [47.1, 88.7]
	South Zone	111	94.0 [69.1, 118.6]
30 second Sit-to-Stand, repetitions	Edmonton	915	2.5 [2.3, 2.8]
	Calgary	922	2.9 [2.7, 3.1]
	North Zone	122	2.8 [2.2, 3.5]
	South Zone	88	3.5 [2.8, 4.3]
Six-minute Walk Test, metres (In-person only)	Edmonton	779	40.0 [35.6, 44.4]
	Calgary	646	37.3 [32.5, 42.1]
	North Zone	86	42.2 [29.2, 55.2]
	South Zone	89	49.9 [37.2, 62.7]
Two-minute Step Test (Virtual Only)	Edmonton	104	12.4 [9.3, 15.5]
	Calgary	245	12.1 [10.0, 14.1]
	North Zone	38	12.2 [7.1, 17.3]
	South Zone	2	-14.7 [-37.0, 7.6]
Combined Grip Strength, kg (In-person only)	Edmonton	781	1.0 [0.5, 1.6]
	Calgary	658	2.4 [1.9, 3.0]**
	North Zone	74	3.0 [1.3, 4.7]
	South Zone	90	4.1 [2.5, 5.6]*
Shoulder Flexion, degrees (Right)	Edmonton	927	1.6 [1.0, 2.2]
	Calgary	933	1.9 [1.3, 2.4]
	North Zone	112	2.8 [1.2, 4.4]
	South Zone	92	4.2 [2.4, 6.0]**
Shoulder Flexion, degrees (Left)	Edmonton	926	1.7 [1.1, 2.3]
	Calgary	931	2.1 [1.6, 2.7]
	North Zone	113	3.2 [1.6, 4.8]
	South Zone	92	4.9 [3.2, 6.7]**
Average One-leg Balance, seconds	Edmonton	847	3.8 [3.2, 4.5]
	Calgary	904	3.8 [3.2, 4.4]
	North Zone	116	4.7 [3.0, 6.4]
	South Zone	74	5.4 [3.3, 7.5]
Lower Body Flexibility, cm (Optional)	Edmonton	810	2.0 [1.6, 2.4]
	Calgary	774	3.1 [2.7, 3.5]**
	North Zone	86	2.8 [1.6, 4.0]
	South Zone	83	7.0 [5.8, 8.2]**
ESAS Total Score	Edmonton	1016	1.2 [0.7, 1.8]
	Calgary	1037	1.2 [0.7, 1.8]

	North Zone	157	-0.7 [-2.1, 0.7]
	South Zone	109	1.0 [-0.7, 2.7]
FACT-G (0-160)	Edmonton	1018	1.8 [1.3, 2.4]
	Calgary	1033	1.6 [1.0, 2.2]
	North Zone	159	0.7 [-0.7, 2.2]
	South Zone	107	1.4 [-0.4, 3.2]
FACIT-Fatigue Scale	Edmonton	1019	2.9 [2.5, 3.4]
	Calgary	1037	2.7 [2.2, 3.1]
	North Zone	159	1.8 [0.6, 3.0]
	South Zone	110	2.7 [1.2, 4.1]
FACT: Trial Outcome Index	Edmonton	1019	4.7 [4.0, 5.5]
	Calgary	1035	4.4 [3.6, 5.2]
	North Zone	159	2.4 [0.5, 4.5]
	South Zone	108	4.3 [1.9, 6.7]
EQ-5D-5L Visual Analogue Scale	Edmonton	1019	5.4 [4.5, 6.2]
	Calgary	1040	5.2 [4.3, 6.0]
	North Zone	159	3.3 [1.1, 5.4]
	South Zone	108	3.3 [0.7, 5.9]

**Controlling for baseline value, age, sex, cancer treatment status, stage/risk status, cancer type;*
***significantly difference in favour of site $p < 0.05$*

Supplementary Table S4. Key Outcomes by Adherence

Outcome	Attendance Category	Sample Size T0 to T1	Adjusted mean difference: T0 to T1 [95% CI]	Difference Between Groups p-value
Physical Activity, minutes (12 weeks)	≥ 80%	1459	85.1 [78.3, 91.9]*	
	< 80%	840	57.7 [48.6, 66.7]	p < 0.001
Physical Activity, minutes (1 year)	≥ 80%	1163	52.6 [44.0, 61.2]	
	< 80%	575	47.7 [35.4, 60.0]	p = 0.526
30 second Sit-to-Stand, repetitions (12 weeks)	≥ 80%	1434	2.9 [2.7, 3.1]*	
	< 80%	609	2.4 [2.1, 2.7]	p = 0.002
FACIT-Fatigue Scale (12 weeks)	≥ 80%	1462	3.6 [3.2, 4.0]*	
	< 80%	845	1.3 [0.8, 1.8]	p < 0.001
EQ-5D-5L Visual Analogue Scale (12 weeks)	≥ 80%	1463	6.1 [5.3, 6.8]*	
	< 80%	846	3.5 [2.6, 4.5]	p < 0.001

**Controlling for baseline value, age, sex, location, cancer treatment status, stage/risk status, cancer type*
